# Supplementary material for: Germination and Simulated Gastrointestinal Digestion of Chickpea (Cicer arietinum L.) in Exhibiting In Vitro Antioxidant Activity in Gastrointestinal Epithelial Cells
Source: Antioxidants (Basel). 2023 May 18;12(5):1114. doi: 10.3390/antiox12051114 (PMC10215366; doi:10.3390/antiox12051114)

Germination and Simulated Gastrointestinal Digestion of Chickpea (*Cicer arietinum* L.) in Exhibiting *in-vitro* Antioxidant Activity in Gastrointestinal Epithelial Cells

Ashley Newton[a], Kaustav Majumder[a#]

[a]Department of Food Science and Technology, University of Nebraska-Lincoln, Lincoln, NE 68588 USA

# Address for correspondence:

Dr. Kaustav Majumder

256 Food Innovation Center, Nebraska Innovation Campus, Department of Food Science and Technology, University of Nebraska-Lincoln, Lincoln, NE, USA, 68588-6205

Telephone: (402)-472-3510; Fax: (402) 472-4474; Email: kaustav.majumder@unl.edu

Supplementary Data

Table S1. Concentration of free amino acids identified in D0-CD or D3-CD samples.

Amino Acid	Day 0 (μg/g sample)[a]	Day 3 (μg/g sample)[a]
Histidine (His)	101.3 ± 17.4	102.2 ± 2.1
Asparagine (Asn)	103.6 ± 6.1	108.4 ± 20.5
Taurine	50.7 ± 5.7	43.9 ± 3.1
Serine (Ser)	80.9 ± 1.6	76.1 ± 7.0
Glutamine (Gln)	163.0 ± 18.8	178.5 ± 8.5
Arginine (Arg)	639.4 ± 41.6	565.9 ± 21.7
Aspartic acid (Asp)	30.9 ± 10.3	38.7 ± 2.1
Glutamic acid (Glu)	105.8 ± 34.9	141.0 ± 7.0
Threonine (Thr)	88.2 ± 8.0	97.0 ± 7.0
Alanine (Ala)	77.9 ± 11.2	90.5 ± 6.8
γ-aminobutyric acid (GABA)	14.9 ± 2.3	21.0 ± 10.6
Proline (Pro)	16.5 ± 16.5	20.2 ± 5.6
Ornithine	8.9 ± 0.7	9.3 ± 0.6
Lysine (Lys)	136.6 ± 30.1	182.4 ± 4.9
Tyrosine (Tyr)	459.1 ± 57.0	404.4 ± 27.5
Methionine (Met)	80.5 ± 13.3	81.2 ± 5.8
Valine (Val)	118.2 ± 17.5	143.3 ± 17.4
Isoleucine (Ile)	126.8 ± 21.7	133.0 ± 13.4
Leucine (Leu)	422.9 ± 41.9	417.3 ± 23.6
Phenylalanine (Phe)	746.8 ± 106.0	675.0 ± 59.8
Tryptophan (Trp)	292.4 ± 22.1	243.0 ± 22.1

[a]Mean ± Standard deviation, SD

Table S2. Total list of identified di- and tripeptides in GCD and their intensity, *m/z*, and retention time (min).

Amino Acid Sequence	Intensity of Day 0[a]	Intensity of Day 3[a]	*m/z*	Retention time (min)
Gly-Asp*	97425 ± 4695	111485 ± 4916	191.0663	11.20
Pro-Asn*	106562 ± 1486	111854 ± 2849	230.1136	10.88
Phe-Glu*	679836 ± 53410	785339 ± 20354	295.1287	9.28
Glu-Phe*	29336070 ± 3758664	17116700 ± 2315890	295.1287	8.24
Glu-Tyr*	5224094 ± 659015	2956915 ± 564178	311.1237	8.83
Leu-Gln*	5533495 ± 405590	6640445 ± 448054	260.1602	3.06
Ile-Lys	6613469 ± 208469	6295463 ± 199190	260.1968	13.77
His-Ile	2797239 ± 259379	3190615 ± 194620	269.1607	13.01
Leu-Val	2493872 ± 337728	3122554 ± 335509	231.1704	8.41
Gly-Ile	718685 ± 514374	626875 ± 426295	189.1231	2.02
Val-Pro	17731 ± 4718	18320 ± 3865	215.1388	1.11
Pro-Thr	9518 ± 258	9097 ± 1078	217.118	4.63
Thr-Pro	198428 ± 27646	223864 ± 19950	217.1181	1.19
Ser-Ile	3692212 ± 582584	4464304 ± 186343	219.1337	2.90
His-Ala	9801 ± 3430	13737 ± 2025	227.1136	2.18
Pyro-Glu-Val	650423 ± 215264	636029 ± 42276	229.1181	3.55
Pro-Ile	1275105 ± 386268	1280836 ± 194307	229.1545	4.03
Pro-Leu	1816590 ± 186541	1947339 ± 202747	229.1545	5.03
Ile-Pro	932919 ± 170656	1002200 ± 114319	229.1545	4.57
Val-Asn	23910 ± 5316	23663 ± 7887	232.1291	1.11
Leu-Thr	1762084 ± 85786	1855138 ± 182021	233.1493	1.34
Thr-Ile	3647724 ± 702508	3608798 ± 522722	233.1493	1.47
Thr-Leu	2415069 ± 637787	2436889 ± 248587	233.1494	4.05
Leu-Thr	4484573 ± 571367	5000543 ± 347108	233.1494	3.48

Pro-Gln	238646 ± 22087	241190 ± 25987	244.1289	1.10
Lys-Pro	157171 ± 7555	143369 ± 12206	244.1653	2.47
Val-Gln	3716505 ± 1541414	3772113 ± 348699	246.1445	1.38
Asn-Ile	5027853 ± 424619	4996072 ± 12448	246.1445	2.95
Leu-Asn	1599642 ± 248726	1637590 ± 67621	246.1445	4.45
Pro-Met	332120 ± 60924	379891 ± 48075	247.1107	2.15
Val-Glu	1391518 ± 135085	1488358 ± 126037	247.1285	1.10
Glu-Val	415136 ± 47257	423218 ± 72978	247.1286	3.01
Ala-Thr	3764302 ± 1046497	4412648 ± 995316	253.1179	1.54
Pro-His	410938 ± 98140	415774 ± 72899	253.1291	4.80
His-Pro	175208 ± 45701	117403 ± 16317	253.1292	5.24
Val-His	13216 ± 1697	12613 ± 726	255.1448	4.60
Ile-Gln	671523 ± 196324	832165 ± 58945	260.1601	2.90
Leu-Lys	60711 ± 31898	57102 ± 7897	260.1965	3.47
Ile-Glu	4783146 ± 1435818	4580444 ± 1098873	261.144	1.67
Leu-Glu	6907591 ± 2010787	6475984 ± 3171277	261.1441	1.91
Leu-Met	4343096 ± 1228530	3514074 ± 259780	263.1413	5.28
Ile-Met	737579 ± 19057	721880 ± 109741	263.1421	5.03
Met-Asp	983531 ± 61254	891863 ± 109618	265.0848	1.77
Val-Phe	3148325 ± 1146242	3013906 ± 958873	265.1542	5.82
Phe-Thr	2618308 ± 444500	2786107 ± 672455	267.1335	2.65
His-Leu	6207 ± 992	7632 ± 1423	269.1604	5.87
Pro-Arg	388767 ± 54629	397463 ± 91425	272.1711	1.98
Met-Glu	435845 ± 42234	492743 ± 53947	279.1004	3.12
Phe-Ile	2022973 ± 522869	2603421 ± 524421	279.1698	6.34
Val-Tyr	2102615 ± 52896	2258202 ± 49527	281.149	4.10

Thr-Tyr	753198 ± 671834	877375 ± 176931	283.1283	2.33
Arg-Asp	54936 ± 7725	60174 ± 10490	290.1456	2.77
Leu-Tyr	684543 ± 203764	825368 ± 214007	295.1647	5.13
Asp-Tyr	273843 ± 64119	250450 ± 79963	297.1075	1.28
Thr-Trp	846861 ± 87264	757935 ± 100896	306.1444	4.37
His-Arg	2272586 ± 190563	2153663 ± 805265	312.1788	6.28
Trp-Gln	24031 ± 2793	26616 ± 5917	333.1533	5.40
Trp-Tyr	17347 ± 1905	20516 ± 4933	368.1601	6.32
Lys-Lys	60601 ± 20445	31045 ± 28521	587.366	15.36
Pro-Val-Lys*	237606 ± 15679	203066 ± 9756	343.2339	14.03
Asp-Lys-Lys*	152507 ± 17243	113261 ± 7917	390.2344	14.59
Ala-His-Arg*	158056 ± 13144	81683 ± 9221	383.2161	5.74
His-Ala-Lys	0	7991 ± 5181	355.2089	8.82
Val-Asp-Lys	275892 ± 78458	264556 ± 51432	361.2075	3.87
Pro-Pro-Arg	2982264 ± 173313	2739620 ± 682047	369.2238	5.01
Glu-Thr-Lys	53625 ± 11932	62424 ± 4723	377.2023	2.20
Thr-Glu-Lys	152836 ± 83893	156875 ± 29859	377.2026	3.77
Glu-Asn-Lys	361196 ± 66697	301114 ± 59688	390.1977	2.31
Asp-Glu-Lys	1539307 ± 255520	1432982 ± 140763	391.1815	1.49
Glu-Val-Phe	63616 ± 6183	88778 ± 17801	394.1968	5.94
Asp-Phe-Arg	418531 ± 191666	174332 ± 44235	437.2137	5.26
Phe-Asp-Arg	184426 ± 93698	217169 ± 24236	437.2138	2.87
Pro-Phe-Arg	37180 ± 1383	24514 ± 6964	837.4721	7.55

[a]Mean ± standard deviation (SD)

*Peptide intensity is significantly different (p < 0.05) from D0-CD or D3-CD samples.

Figure S1. Peptides identified in D0-CD and D3-CD samples.

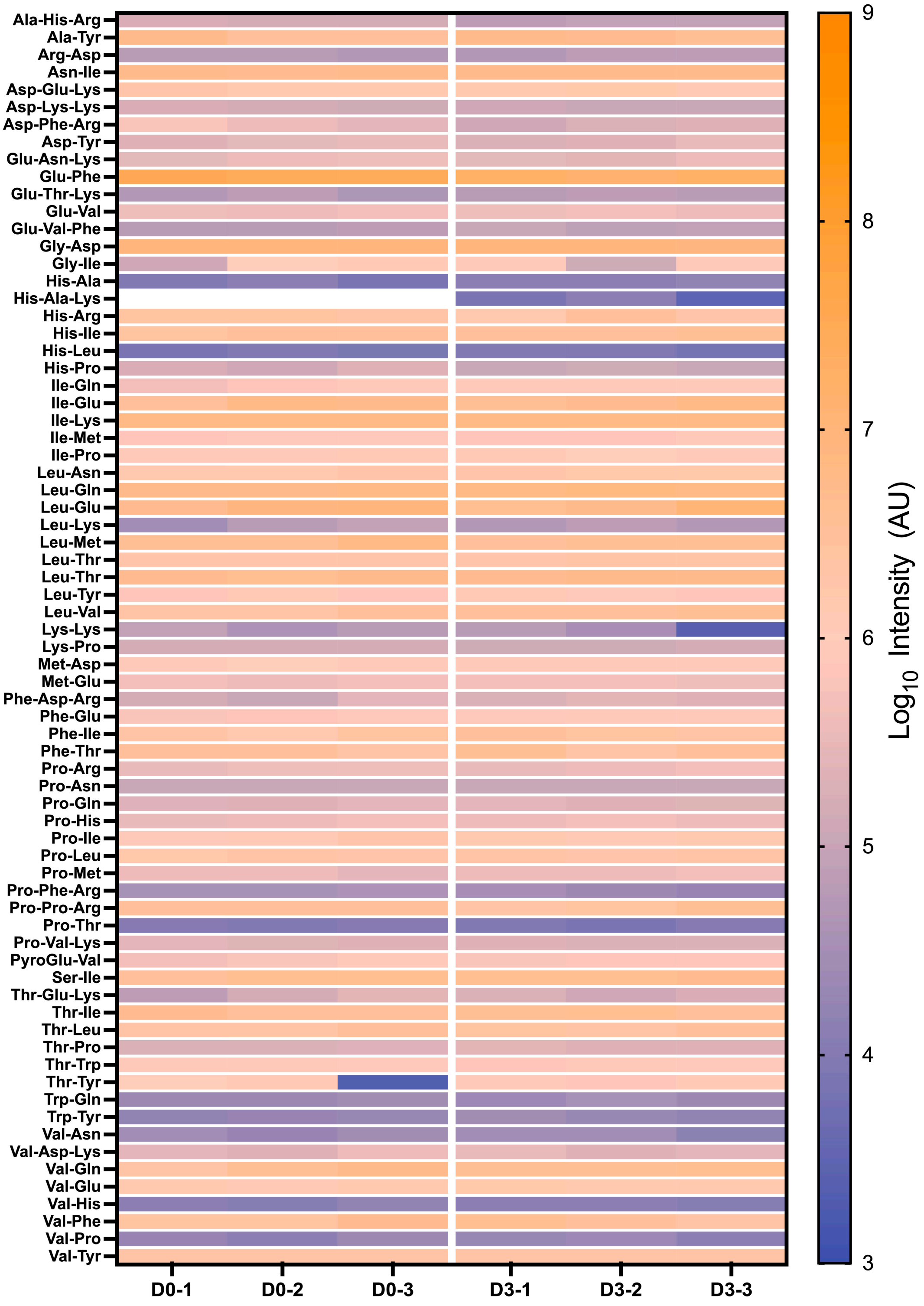

Supplement: Supplementary file 1 [file antioxidants-12-01114-s001.zip › antioxidants-2318415-supplementary.pdf]
